# Molecular Mechanisms and Management of a Cutaneous Inflammatory Disorder: Psoriasis

**DOI:** 10.3390/ijms18122684

**Published:** 2017-12-11

**Authors:** Yu Ri Woo, Dae Ho Cho, Hyun Jeong Park

**Affiliations:** 1 Department of Dermatology, Yeouido St. Mary’s Hospital, College of Medicine, The Catholic University of Korea, Seoul 07345, Korea; w1206@naver.com; 2 Department of Life Science, Sookmyung Women’s University, Seoul 04310, Korea; cdhkor@sookmyung.ac.kr

**Keywords:** biologics, epigenetics, genetics, interleukin-23, psoriasis, signaling pathway, small molecules, T helper 17 cells

## Abstract

Psoriasis is a complex chronic inflammatory cutaneous disorder. To date, robust molecular mechanisms of psoriasis have been reported. Among diverse aberrant immunopathogenetic mechanisms, the current model emphasizes the role of Th1 and the IL-23/Th17 axis, skin-resident immune cells and major signal transduction pathways involved in psoriasis. The multiple genetic risk loci for psoriasis have been rapidly revealed with the advent of a novel technology. Moreover, identifying epigenetic modifications could bridge the gap between genetic and environmental risk factors in psoriasis. This review will provide a better understanding of the pathogenesis of psoriasis by unraveling the complicated interplay among immunological abnormalities, genetic risk foci, epigenetic modification and environmental factors of psoriasis. With advances in molecular biology, diverse new targets are under investigation to manage psoriasis. The recent advances in treatment modalities for psoriasis based on targeted molecules are also discussed.

## 1. Introduction

Psoriasis is a chronic relapsing cutaneous inflammatory disorder. The skin lesion of psoriasis is classically characterized by erythematous papules and plaques with white thick scales. Clinical phenotypes of psoriasis include psoriasis vulgaris, guttate psoriasis, inverse psoriasis, erythrodermic psoriasis and pustular psoriasis [1]. Different clinical phenotypes of the disease can be observed even in the same patients in the course of the disease progression.

The prevalence rate of psoriasis varies in different populations, ranging from 0.91–8.5% [2]. Enamandram et al. [3] surmised that the reason for such diverse prevalence rates of psoriasis among different populations might be associated with genetic susceptibility and environmental factors.

Many hypotheses have been suggested to identify possible pathogenic models for psoriasis. Since the clinical characteristic of psoriasis is manifested as thick scales with the histopathological characteristic of epidermal hyperplasia, psoriasis has been regarded as a disorder of keratinocytes. However, increased discoveries in the pathophysiology of psoriasis have revealed that perturbed differentiation and proliferation of keratinocytes might be due to the interplay between immune cells and keratinocytes in a genetically-susceptible patient. In this review, recent progress in molecular mechanisms of psoriasis that contribute to the initiation and development of psoriasis will be described, especially in the field of immunologic abnormalities, signaling pathways, genetics and epigenetics. New promising treatment targets for psoriasis based on these associated molecular pathways of psoriasis will also be discussed.

## 2. Immunological Abnormalities

### 2.1. The Roles of Adaptive T Cell Immunity and the IL-23/Th17 Axis in Psoriasis

**Figure 1:**
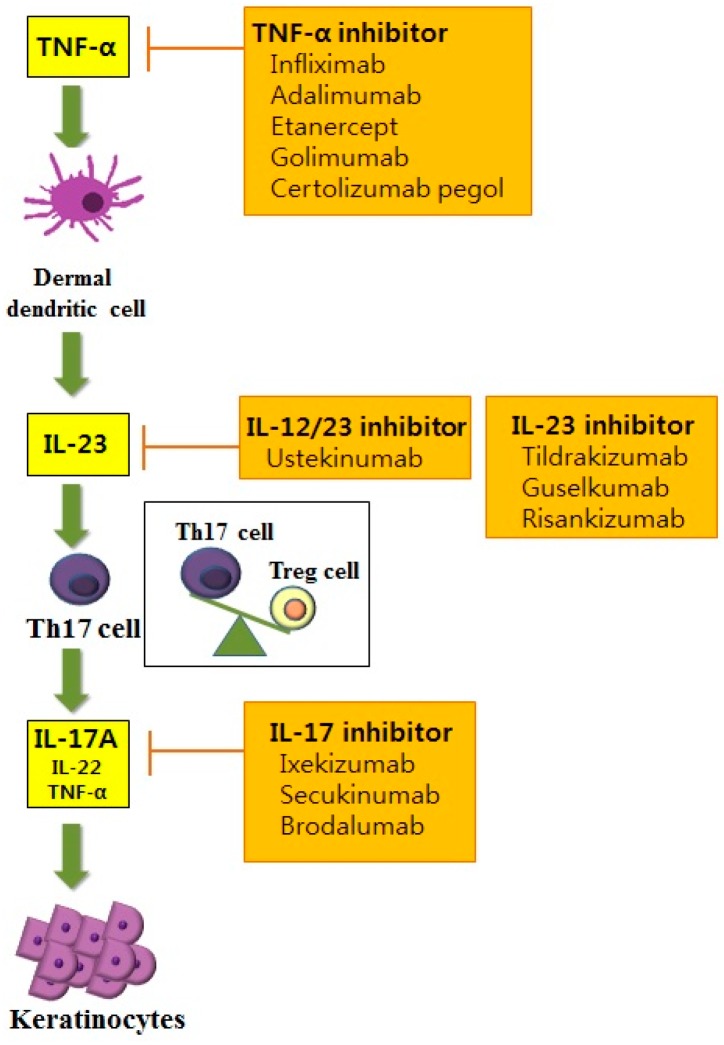
The prevailing model of the IL-23/Th17 axis in the immunopathogenesis of psoriasis and newly-developed targeting molecules in psoriasis. TNF, tumor necrosis factor; Th, T helper cell; Treg, regulatory T cell; IL, interleukin.

Th cells have long been recognized as a key pathogenic factor in psoriasis. The majority of infiltrating CD4^+^ T cells in psoriasis are Th1 cells characterized by their production of Th1 cytokines such as interferon-γ (IFN-γ), interleukin (IL)-2 and IL-12. Elevated serum levels of Th1 cytokines have been observed in the sera of patients with psoriasis. They are also correlated with the severity of psoriasis [4]. Among various Th1 cytokines, the role of IFN-γ in psoriasis has been postulated as an activator of monocytes, dendritic cells (DCs) and endothelial cells [5]. In addition, IFN-γ affects keratinocytes by inhibiting apoptosis [5], suggesting its role in inducing the hyperproliferation of keratinocytes that is observed in psoriasis. However, anti-IFN-γ therapy for managing psoriasis has produced unfavorable results, implying that other pathways are more closely related to the pathogenic mechanisms of psoriasis [6]. With the advances of research in the immunopathogenesis of psoriasis, a recent pathogenic model for psoriasis has postulated the importance of the IL-23/Th17 immune axis (Figure 1). Among the diverse molecules related to this axis, tumor necrosis factor (TNF)-α, IL-23, IL-17 and IL-22 have been established as the key regulators of psoriasis based on the profound effects of biologics targeting these molecules.

The increased expression of IL-23 and its receptor has been observed in the lesional skin in patients with psoriasis compared to that of the normal controls [7]. IL-23 is secreted by DCs and macrophages and stimulates the differentiation and proliferation of the Th17 cell. Thereby, IL-23 serves as an upstream regulatory cytokine in psoriasis. IL-23 has a potent role for activating Th17 cells to release IL-17, IL-22 and TNF-α, which are associated with further inflammatory responses [8]. Moreover, IL-23 induces the production of TNF-α from macrophages. This cytokine is also capable of stimulating hyperproliferation of keratinocytes. Indeed, direct dermal injection of IL-23 can induce epidermal hyperplasia commonly observed in skin samples of psoriasis patients [9].

Stimulated by IL-23, Th17 cells primarily secret IL-17. Besides Th17 cells, a recent study demonstrated the production of IL-17 in neutrophils and mast cells [10]. The IL-17 cytokine family consists of six subtypes (IL-17A–F) [11]. Among them, IL-17A is a key molecule for amplifying inflammation in psoriasis through the propagation of the production of various proinflammatory cytokines and chemokines [11]. Especially, IL-17A can activate keratinocytes to express various proinflammatory cytokines including IL-6, IL-8 and TNF-α and chemokines including CCL20, CXCL1, CXCL2, CXCL3, CXCL5 and CXCL8. IL-17A induces the increased expression of antimicrobial peptides including β-defensin and S100A families, thereby activating the innate immune system [12]. IL-17A can also potentiate the hyperproliferation of keratinocytes and inhibit the differentiation of keratinocytes [10]. Rizzo et al. [13] have injected recombinant murine IL-23 into the skin of IL-17A knockout mice and found little epidermal hyperplasia. In patients with psoriasis, the elevated expression of IL-17 mRNA has been found in skin from patients with psoriasis compared with healthy controls [14]. Moreover, the serum levels of IL-17A are positively correlated with the severity of psoriasis [4].

Besides IL-17A, Th17 cells also secret IL-22. Recently, the new subset of effector T cells that secrete IL-22 was discovered and named Th22 cells. Like Th17 cells, the differentiation and maintenance of Th22 cells are also affected by IL-23 [15]. In patients with psoriasis, the serum levels of IL-22 are significantly elevated in patients with psoriasis compared to normal controls [16]. IL-22 can increase the expression of antimicrobial peptides that are elevated in psoriasis patients in cooperation with IL-17 [12]. IL-22 can also augment the expression of cytokines and chemokines and synergistically regulate downstream effector responses associated with the Th17 pathway [17]. In addition, IL-22 can influence epidermal hyperproliferation and differentiation of keratinocytes [18]. Indeed, in an in vivo model, IL-22 mediates the IL-23-associated keratinocyte hyperproliferation via STAT3 signaling, implicating the involvement of an interplay between two cytokines [19]. A recent study has found that mice deficient in IL-22 binding protein (IL-22BP) show aggravation of psoriasis with increased expression of IL-22-associated antimicrobial peptides [20]. Injection of IL-22BP neutralizing antibody, a natural IL-22 inhibitor, also reproduces the exacerbation of skin inflammation found in the mouse model of psoriasis [20]. Moreover, the ratio of IL-22/IL-22BP is positively correlated with psoriasis severity [20], implying the influence of IL-22 in pathogenetic immune circuits of psoriasis.

It is important to know that the efficacy of Th17 cytokines on the formation of the psoriatic lesion is influenced by these cytokines and by inflammatory mediators such as TNF-α. TNF-α can be produced by DCs, activated T cells, macrophages, Langerhans cells and keratinocytes [21]. Increased TNF-α levels are found in various chronic inflammatory disorders including psoriasis, psoriatic arthritis and rheumatoid arthritis [22,23]. In addition to its proinflammatory effects, the pivotal effects of TNF-α on the pathogenesis of psoriasis are exerted through the regulated production of IL-23 from DCs. Thus, TNF-α modulates the key pathway of the IL-23/Th17 axis in psoriasis. In addition, reduced expression of co-stimulatory factors including CD86 and CD11c was observed in lesional DCs after treatment with TNF-α inhibitor [24]. Moreover, treatment with TNF-α inhibitor can result in the reduced expression of IL-17 and IL-22 on the lesional psoriatic skin in patients with psoriasis. These observations indicate that the disturbed interaction between DCs and T cells after exposure to TNF-α inhibitor might result in the reduced expression of IL-23 and decrease the expression of Th17 effector cytokines, including IL-17 and IL-22. Besides upstream regulation of IL-23, TNF-α can act synergistically with IL-17A on epidermal keratinocytes, resulting in overexpression of various psoriasis-related proinflammatory gene including S100A7, IL-8, DEFB4, CCL20 and CXCL1 [25].

Since perturbed IL-23/Th17 pathways can promote chronic skin inflammation and epidermal hyperplasia in psoriasis, targeting this pathway is attracting much interest [17]. In addition to Th17 cells and their associated molecules, the role of regulatory T (Treg) cells and their association with Th17 cells has been identified in psoriasis. Treg cells are characterized by their expression of CD4, CD25 and forkhead/winged helix transcription factor 3 (FoxP3) [26]. Treg cells play an important role in suppressing inflammation of other immune cells such as T cells, thereby affecting homeostasis of the immune system. Various chronic inflammatory diseases show dysregulation in Treg cells, including rheumatoid arthritis, atopic dermatitis, pemphigus vulgaris and psoriasis [27,28,29,30]. Infiltration of both Th17 cells and Treg cells is increased in patients with psoriasis [26]. In addition, the ratio of infiltrating Th17 cells to Treg cells on psoriatic skin is positively correlated with the severity of psoriasis. The activity of Treg cells in peripheral blood is decreased in patients with psoriasis, and the number of Treg cells in psoriatic skin lesions is normal [30]. However, the same study found that the capability of Treg cells suppressing CD4^+^ T cells is impaired in patients with psoriasis [30]. Therefore, the impaired immunoregulatory function of Treg cells in psoriasis might propagate excessive T cell proliferation, further aggravating inflammatory reactions.

### 2.2. Role of Skin Resident Cells in Psoriasis

#### 2.2.1. Keratinocytes in Psoriasis

Although the pathogenesis of psoriasis has long been explained on the basis of perturbance in T cell populations, keratinocytes also play an important role in the activation of certain T cell populations by expressing various autoantigens. Keratinocytes could assist activation of T cells upon exposure to bacterial, viral or fungal pathogens in psoriasis. Especially, streptococcal superantigen is a major autoantigen in psoriasis. The production of streptococcal superantigen requires T cell presentation to MHC class II molecules expressed on keratinocytes [31]. In addition, the overexpression of antimicrobial peptides LL-37 produced from keratinocytes has also been observed in psoriatic skin [32]. LL-37 acts as an autoantigen and stimulates the activation of T cells in psoriatic patients.

In addition, an imbalance between differentiation and proliferation of keratinocytes occurs in patients with psoriasis [33]. Lesional psoriatic epidermal keratinocytes upregulate K6/K16, which is a marker for hyperproliferation [33,34]. The downregulation of K1/K10, a marker for terminal differentiation, in the suprabasal layer has also been observed in the suprabasal layer in the lesional epidermal skin in patients with psoriasis [33,34]. The accelerated proliferation of basal keratinocytes and differentiation of spinous and granular keratinocytes are the key features observed in psoriasis and wound repair [35]. Therefore, this process is called regenerative maturation [36]. During the process of regenerative maturation, altered expression levels of genes including CDSM and genes encoding EDC have been found in keratinocytes of psoriatic plaques [35]. In addition, expression of proteins, such as S100A9 and S100A9, associated with regeneration of epidermal keratinocytes also functions as chemotactic factors for neutrophils in psoriasis [35]. The cytokines released from neutrophils, such as TNF-α and IFN-γ, could promote the production of adhesion molecules and chemokines in keratinocytes and could intensify the recruitment of neutrophils in epidermal keratinocytes [35]. Therefore, the pathways involved in regenerative maturation in psoriasis are associated with activation of complex dysregulated immunological circuits.

Keratinocytes from patients with psoriasis have aberrant responses for the production of proinflammatory cytokines, including IL-1, IL-8 and IL-15 [37]. Especially, the dysregulation in IL-1 receptor signaling has been observed in the lesional skin of patients with psoriasis [38]. The epidermal barrier disruption frequently occurs in psoriasis, resulting in the increased expression of IL-1 and IL-1 receptor antagonists [33]. Along with proinflammatory cytokines, diverse effector molecules including growth factor, chemokines, eicosanoids and antimicrobial peptides are released from keratinocytes and augment the inflammatory responses in psoriasis [37,39,40]. The regulation of growth in psoriatic keratinocytes is mainly controlled by various growth factors, including epidermal growth factor (EGF) and keratinocyte growth factor (KGF) [33]. The members of the EGF family such as TGF-α and amphiregulin modulate the growth of epidermal keratinocytes in an autocrine fashion [41]. In an experimental model, the overexpression of EGF and its receptor induces the psoriasis-like skin hyperplasia and inflammation [42]. In addition, the activation of the EGF receptor further induces the production of vascular endothelial growth factor in keratinocytes, suggesting a relevant role in the growth of epidermal keratinocytes and angiogenesis observed in psoriasis [43]. Expression of KGF was recently observed in the lesional epidermal skin of a patient with psoriasis [44]. In a recent study, the expression of the KGF receptor was positively correlated with the degree of proliferation of HaCa T keratinocytes [45]. KGF also promotes the expression of α5ß1 integrin and inhibits the expression of K10 in keratinocytes [46]. Concerning chemokines, production of diverse chemokines from epidermal keratinocytes might considerably affect the activation of inflammatory cells in psoriasis. For example, chemokines like MIG/CXCL9, IL-10/CXCL10, IL-8/CXCL8, MCP-1/CCL2 and MIP-3α/CCL20 released from epidermal keratinocytes function as potent chemoattractants for monocytes, neutrophils, Langerhans cells, DCs and T cells, which are key cells in psoriasis [31,47]. Moreover, non-lesional skin of the patients with psoriasis also releases keratinocyte-derived factors such as extra domain A-positive fibronectin; thereby, keratinocyte could modulate the autocrine circuit resulting in the epidermal hyperplasia in psoriasis [33]. All of these observations demonstrated that the psoriatic keratinocytes exert a great influence on the regulation of inflammatory cascade in psoriasis, and unraveling the complex immunologic circuits in psoriatic skin merit further investigations.

#### 2.2.2. Other Skin-Resident Immune Cells in Psoriasis

Skin is also a sentinel for the immune system. The major role of skin as a peripheral lymphoid organ has been determined due to skin-associated lymphoid tissues [48]. Circulating skin-homing T cells express cutaneous lymphoid antigen and chemokine receptor (CCR)4, CCR6 and CCR10 [49,50]. However, the vast majority of cutaneous lymphoid antigen-positive T cells are residents of normal skin [51]. In addition to skin-homing T cells, other cells, including natural killer (NK) cells, natural killer T, DCs and Langerhans cells, are also found in normal skin [50,52,53]. A mouse model in which symptomless psoriatic skin was engrafted in severely immunodeficient mice lacking functional NK cells, T cells or B cells revealed the spontaneous production of psoriatic skin lesions, suggesting that tissue-resident immune cells themselves can be a major source for the development of psoriasis [54]. However, some researchers have argued that the aforementioned mouse model cannot fully represent a psoriatic model as it is not proper for evaluating the properties of leukocyte recruitment in psoriasis [50]. Moreover, patients with psoriasis treated with allogeneic bone marrow transplantation show resolution of their skin lesions, implying that not only skin-resident immune cells, but also systemic immune cells are important for the development and maintenance of psoriasis [55].

## 3. Major Signal Transduction Pathway Alterations in Psoriasis

The signal transduction pathway is another key regulator of various immune and inflammatory disorders and is involved by adjusted proliferation, differentiation and apoptosis of the cells. The complex nature of psoriasis pathogenesis can also be explained by altered signal transduction pathways. To date, altered signal transduction pathways have been observed in psoriasis including nuclear factor-kappa B (NF-κB), Janus kinase-signal transducers and activators of transcription (JAK-STAT), Akt and Wnt pathways. The dysregulation in these pathways influences the activation and trafficking of immune cells. In addition, these signal transduction pathways also regulate the survival, proliferation and differentiation of keratinocytes in psoriasis. Various studies have confirmed the dysregulation of signal transduction pathways in psoriasis using in vitro analyses, animal models and genomic analyses, as well as psoriatic lesional skin biopsies. In this section, the current knowledge of major signal transduction pathways involved in the pathogenesis of psoriasis will be discussed.

### 3.1. NF-κB Signaling Pathways

**Figure 2:**
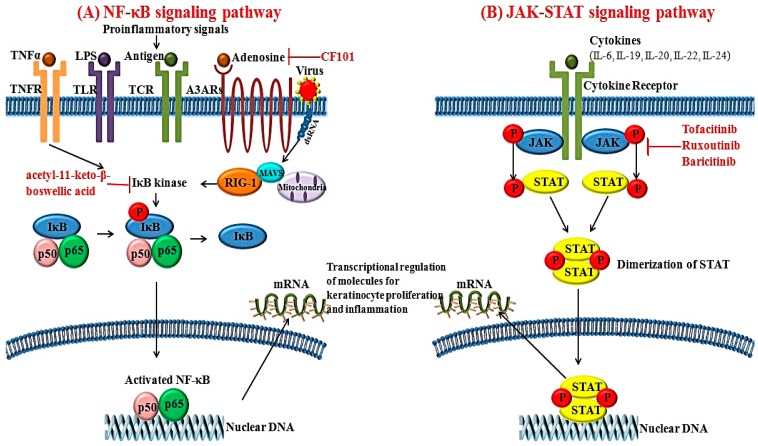
Schematic view of (**A**) the NF-κB signaling pathway and (**B**) the JAK-STAT signaling pathway that contribute to the pathogenesis of psoriasis and its novel targeting molecules. T-bar implies inhibition; The circled P represents phosphorylation; NF, nuclear factor κB; TNF, tumor necrosis factor; LPS, lipopolysaccharide; TLR, toll-like receptor; TCR, T-cell receptor; A3AR, A3 adenosine receptor; RIG-1, retinoic acid inducible-gene 1; JAK, Janus kinase; STAT, signal transducers and activators of transcription.

The activated form of NF-κB is a heterodimeric transcription factor composed of the p65 and p50 subunits [56]. After IκB kinase induces the detachment of NF-κB from IκB, NF-κB enters the nucleus and affects specific sequences of target genes [56]. Various stimuli can activate NF-κB signaling pathways; these include TNF-α, IL-1, IL-17, virus and lipopolysaccharide [57]. In psoriasis, expression of NF-κB is upregulated in the psoriatic skin compared to healthy controls [58]. As a pivotal element for regulating the inflammatory process, NF-κB can modulate the transcription of various cytokines, chemokines, adhesion molecules and enzymes. It also affects the production of inflammatory cytokines such as TNF-α, IL-1, IL-6 and IL-8 (Figure 2A). In addition, NF-κB activation can affect the differentiation and proliferation of keratinocytes in psoriatic skin [59].

Recently, increased expression of retinoic acid inducible-gene 1 (RIG-1) was reported in psoriatic skin lesions [60]. RIG-1 is a major sensor for RNA viruses and activates NF-κB signaling pathways [60]. Elevated expression of RIG-1 has also been observed in IL-23 and an imiquimod-induced mouse model of psoriasis [60]. RIG-1-deficient mice show lower degrees of epidermal hyperplasia and inflammatory cell infiltration. The gene that encodes RIG-1 is *DDX58*, a well-known susceptibility gene for psoriasis [61]. Zhu et al. [60] have found that activation of NF-κB signaling pathways via RIG-1 is associated with the production of IL-23 in DCs in psoriasis. Based on these findings, RIG-1 could be another novel target for managing inflammation and keratinocyte hyperproliferation in psoriasis.

### 3.2. JAK-STAT Signaling Pathways

JAK is a cytoplasmic tyrosine kinase important for inducing cytokine-associated signaling pathways [11]. To date, four subtypes of JAKs have been identified: JAK1, JAK2, JAK3 and tyrosine kinase 2 [11]. Activation of JAK can be initiated by various cytokines and growth factors. Activated JAK will phosphorylate STAT. Therefore, JAK has effects on both the signal transducer and transcription factor (Figure 2B) [62]. Among the various STATs, STAT3 can modulate cellular proliferation, differentiation and apoptosis. Cytokines including IL-6, -19, -20, -22 and -24 have been implicated in the pathogenesis of psoriasis and can initiate the activation of STAT3 [63]. In fact, STAT3 expression is upregulated in the psoriatic skin lesions compared to healthy controls [64]. Moreover, transgenic mice overexpressing active STAT3 cDNA can exhibit spontaneous psoriatic skin lesions [64].

## 4. Genetics

Genetic studies for psoriasis have offered pivotal pathogenic insights regarding psoriasis pathogenesis. The earlier case-control studies have been conducted to identify the genetic association between major histocompatibility complex (MHC) and psoriasis [65,66,67]. Tiilikainen et al. [67] found that the prevalence of HLA-Cw6 was 45.9% in patients with psoriasis vulgaris and 7.4% in controls. A higher prevalence of human leukocyte antigen (HLA)-B13-positive cases among patients with psoriasis was observed compared to control phenotypes [68]. However, among diverse HLA alleles, specific HLA alleles alone are not adequate for the development of psoriasis, which could imply that other genes are involved in psoriasis pathogenesis [69].

To find out more specific candidate genes for psoriasis, classical linkage studies have been conducted. To date, 13 psoriasis susceptibility locus (PSORS)1-13 have been identified to be associated with psoriasis [70]. PSORS1 is located on chromosome 6p 21.3. It is the most frequently-replicated locus for psoriasis [71]. PSORS1 contains genes encoding *HLA-C* and corneodesmosin [72]. However, the possible occurrence of linkage disequilibrium across the suspected region caused the determination of specific disease susceptibility allele challenging. To better localize the psoriasis susceptibility locus, researchers used linkage disequilibrium mapping. For example, linkage disequilibrium mapping revealed that the PSORS1 is located in the proximity of HLA-C, which could cause the narrowing of the critical interval for the psoriasis susceptibility gene [73,74,75]. For other PSORS, PSORS2 on chromosome 17q25.3 has been found to contain genes encoding *CARD14*, *SLC9A3R1*, *NAT9*, *RAPTOR* and *TBCD* [76,77], while PSORS4 on 1q21.3 has been found to be located within the epidermal differentiation complex (EDC) which contains *Loricrin*, *Filaggrin*, *Pglyrp* and *S100* genes [78]. However, many other PSORS found from linkage analyses have not been replicated, except for PSORS1, PSORS2 and PSORS4 [71].

With the development of genome-wide association studies (GWAS), specific alleles associated with a disease could be easily identified. In a GWAS study, markers for millions of single nucleotide polymorphisms are analyzed for their allelic differences between cases and controls [71]. Recently, various large-scale GWASs for psoriasis have identified possible risk factors for psoriasis (Table 1). The susceptibility loci for psoriasis contain many genes associated with disease pathogenesis such as genes involved in antigen presentation, Th1 cell differentiation, Th17 cell differentiation, nuclear factor κB (NF-κB) signaling, IFN signaling and keratinocyte proliferation. In relation to adaptive immunity, the genetic locus that provides the strongest association for psoriasis susceptibility is MHC class I [79,80]. Among MHC molecules, HLA-Cw6 on 6p 21.3 provides the strongest association with psoriasis [81,82]. Endoplasmic reticulum aminopeptidase 1 (*ERAP1*) is another well-known gene associated with the genetic risk locus for psoriasis [83]. ERAP protein functions as a peptidase that modulates the binding of peptides to the MHC class 1 molecule [71]. Genetic variants of *ERAP1* alleles can affect their interactions with HLA-Cw6 [83]. This provides further insights into the pathogenesis of psoriasis resulting from genetic variants related to antigen presentation to an adaptive immune system. In addition, *RUNX3* has been found to be associated with T helper (Th) 1 cell differentiation in psoriasis [84,85]. Moreover, *SOC1*, *IL-12B*, *IL-23R*, *TRAF3IP2* and *IL23A* have been found to be related to the pathway involved in Th17 cell differentiation in psoriasis [79,86,87,88]. The role of innate immunity in the pathogenesis of psoriasis has been increasingly emphasized [89], and genetic variants associated with psoriasis have been increasingly explored. Genes *IFIH1*, *DDX58*, *RNF114*, *IL-28RA*, *TYK2*, *EXOC2* and *ELMO1* have been found to be involved in IFN-mediated antiviral pathway of psoriasis [61,83,90,91,92,93]. Genes *CARD14*, *REL*, *TNIP1/ANXA6*, *TNFAIP3*, *UBE2L3*, *CARM1*, *NFKBIA* and *FBXL19* have been revealed to be involved in the NF-κB signaling pathway of psoriasis [61,84,89,91,94,95,96,97]. Recently, the largest meta-analysis of GWAS for psoriasis has identified 16 novel genetic loci for psoriasis [94], including *CHUK* on chromosome 10q24.31, *FASLG* on chromosome 1q24.3 and *IKBKE* on chromosome 1q32.1, which are associated with the NF-κB signaling pathway. In addition, genes for regulation of the skin barrier within EDC have been proven to be associated with psoriasis. A recent GWAS in the Chinese population found a relationship between psoriasis and SNPs in the *LCE* gene cluster [98]. Moreover, De Cid et al. have revealed that deletion of *LCE3B* and *LCE3C* is associated with higher risk for psoriasis in the European population [99].

Although GWAS studies can be exerted as a potent tool for identifying the association between genes and psoriasis, there are some potential limitations. Rare genetic variations are difficult to detect through GWAS studies [103]. Moreover, the possibility of the occurrence of linkage disequilibrium and population stratification might bias the experimental results [104]. With the advent of next generation sequencing, recognition of rare alleles can be more easily investigated than before. Recently, Tang et al. [103] explored the single nucleotide variants associated with psoriasis in *GJB2*, *IL23R*, *ERAP1*, *LCE3D*, *ERAP1*, *ZNF816A* and *CARD14* among the Han Chinese population by next generation sequencing. Further studies using next generation sequencing technology in psoriasis are needed and are expected to lead to the increased understanding of the genetics of psoriasis.

## 5. Epigenetics

Dysregulation in the epigenetic network has been suggested to be one possible pathogenic factor in various autoimmune disorders, including atopic dermatitis, systemic lupus erythematosus, rheumatoid arthritis, systemic sclerosis and psoriasis [105]. In twin studies, the concordance rate in psoriasis among monozygotic twins has been found to be 36–64% [106,107]. Such discordance in monozygotic twins implies that epigenetic modification might also play an important role in the development of psoriasis. DNA methylation, histone modification and microRNA (miRNA) profile are the major three epigenetic modifications in psoriasis. The emerging role of epigenetic modifications in psoriasis will be described below.

### 5.1. DNA Methylation

DNA methylation in psoriasis occurs both in lesional skin of psoriasis and peripheral blood mononuclear cells (PBMCs). Furthermore, it regulates the gene expression [108]. DNA methylation frequently occurs in cytosine-guanine islands within gene promoter lesion [109]. Cytosine-guanine hypomethylation in p15/CDKN2B and p21/CDKN1A genes involved in cell cycling has been observed in mononuclear cells isolated from bone marrow of patients with psoriasis [110]. In addition, the number of highly-proliferative potential colony-forming cells in bone marrow of psoriasis patients is significantly decreased compared to that in the normal control group [110]. As psoriasis is characterized by prominent epidermal hyperproliferation, it has been supposed that keratinocytes in lesional skin samples of patients with psoriasis show resistance to programmed cell death [111]. The p16^INK4a^ promoter region is as an anti-apoptotic molecule and was shown to be hypermethylated in 30% of psoriasis patients [112]. Psoriasis Area and Severity Index (PASI) scores were also elevated in patients with hypermethylation of p16^INK4a^ [112]. Recently, Zong et al. [113] have reported that HLA-DRB1 is hypomethylated in lesional skin samples of patients with psoriasis and is negatively correlated with PASI scores.

### 5.2. Histone Modification

In addition to DNA methylation, histone modification, which occurs through acetylation, deacetylation, phosphorylation, ubiquitination, deamination or proline isomerization tag, is associated with psoriasis [114]. Acetylation and deacetylation of histone tails are regulated by histone acetyltransferases and histone deacetylases (HDACs) [115]. Hypoacetylation of global histone H4 has been observed in PBMCs of psoriasis patients compared to that in normal controls. In addition, the degree of hypomethylation is negatively correlated with PASI score [115]. Moreover, overexpression of HDAC-1 mRNA has been observed in skin samples from psoriasis patients. It might cause overexpression of hypoxia inducible factor-1α in hypoxic conditions [116]. Therefore, the HDAC inhibitor has emerged as a promising agent for controlling immune and inflammatory diseases, including psoriasis [117,118].

### 5.3. miRNAs

Recently, various studies have been conducted to determine the role of miRNAs in the pathogenesis of inflammatory skin disorders such as atopic dermatitis [119] and psoriasis [120]. miR-125b regulates proliferation and differentiation of keratinocytes by regulating functions of fibroblast growth factor receptor 2. Downregulation of miR-125b has been observed in the lesional skin of patients with psoriasis and is negatively correlated with expression levels of fibroblast growth factor receptor 2 [121] and TNF-α [122]. In addition, expression of miR-146a has been found to be increased in lesional skin samples of patients with psoriasis. miR-146a modulates IL-17-associated skin inflammation in human skin and in a mouse model and is also involved in the TNF-α signaling pathway [115,123,124]. In a recent study, Hermann et al. [120] have found that miR-146b and miR-146a are overexpressed in lesional skin samples of patients with psoriasis. FERMT1 associated with keratinocyte hyperproliferation is the direct target gene for miR-146a. [120]. Therefore, increased expression of miR-146a and miR-146b in psoriasis might be a mechanism underlying the proliferation of keratinocytes in psoriasis. Recently, Wang et al. [125] have reported that the miRNA axis is imbalanced in psoriasis. In addition to miR-146 and miR125b, the authors identified other miRNAs associated with psoriasis, including miR-31, miR-203 and has-miR-99a [125]. They suggested that increased expression of miR-31 and miR-203 with decreased expression of has-miR-99a and miR-125b might contribute to the imbalance in the miRNAs axis in psoriasis [125]. Indeed, various molecules associated with diverse miRNA modifications in psoriasis have been identified. Key miRNAs associated with psoriasis are summarized in Table 2. Further studies are needed to reveal interconnected epigenetic modifications in psoriasis.

### 5.4. Long Noncoding RNA

Long non-coding RNAs (lncRNAs) could be defined as non-protein coding transcripts that are longer than 200 nucleotides [141]. Recent studies found that the lncRNAs exert an important role in regulating the immune system by modifying gene expression in diverse inflammatory diseases such as systemic lupus erythematosus, rheumatoid arthritis and psoriasis [142,143,144]. The expression of lncRNA gene *PRINS* was elevated in the non-lesional skin of patients with psoriasis compared to the lesional skin of patients with psoriasis and healthy controls, implying that *PRINS* plays a role as a psoriasis susceptibility gene [144]. In addition, the other lncRNA gene, *PSORS1C3*, which resides within PSORS1, has been identified as an important psoriasis susceptibility gene in Swedish and Chinese populations [145,146]. Although various studies have been conducted to identify the association between inflammatory disorder and specific lncRNA genes, in fact, a small number of lncRNA genes has been found in psoriasis. Further studies are required to find more associated lncRNA genes and to identify possible interrelationships of these lncRNA genes with other epigenetic modifications in psoriasis.

## 6. Environmental Factors

### 6.1. Obesity

A variety of epidemiological studies has identified the relationship between psoriasis and obesity. Wolk et al. [147] found that obese individuals have a greater risk of having psoriasis more than twice as much as healthy controls. Moreover, the prevalence and severity of psoriasis are positively correlated with the presence of obesity [148,149]. The adipose tissue itself functions as an important endocrine, paracrine and autocrine organ via various adipokines and inflammatory cytokines [148]. Moreover, the composition of adipose tissue is altered in obese people, which potentiates further proinflammatory responses [148]. Among various molecules, the major cytokines that are concurrently expressed in both adipose tissue and skin of patients with psoriasis are IL-6 and TNF-α [148]. The simultaneously increased expressions of inflammatory cytokines observed in both conditions might provide a potential link to the pathogenic mechanisms between obesity and psoriasis.

### 6.2. Alcohol Consumption

Alcohol is a potent environmental risk factor for psoriasis. Qureshi et al. [150] revealed that heavy alcohol consumption and development of psoriasis are positively correlated. Moreover, patients with moderate to severe psoriasis tend to have a more increased incidence of alcohol-related diseases such as depression and cardiovascular diseases [151]. The metabolites of alcohol increase the expression of soluble TNF-receptor type 1 and TNF-α converting enzyme, which are associated with propagating inflammation in psoriasis [152]. In addition, the effects of ethanol and acetone on HaCa T keratinocytes were explored in an in vitro model [153]. The proliferative response in HaCa T cells was observed after treatment with acetone and ethanol [153], suggesting that alcohol consumption might affect the epidermal hyperproliferation observed in psoriasis.

### 6.3. Psychological Stress

Psychological stress also plays a potent role in the initiation and aggravation of psoriasis [154]. In patients with psoriasis, psychologically-stressful conditions resulted in the upregulation of sympathetic-adrenomedullary system and downregulation of the hypothalamus-pituitary-adrenal axis [155,156]. In these circumstances, decreased levels of cortisol and increased levels of epinephrine and norepinephrine can be observed in patients with psoriasis when compared with healthy controls [156]. The altered levels in these values in patients with psoriasis further leads to the degranulation of mast cells, alteration in skin barrier function and the release of proinflammatory cytokines, all of which are associated with the pathogenesis of psoriasis [157]. Moreover, patients with psoriasis suffer from stress due to the disease itself, which can cause a chronic disfiguring appearance of the patient [155]. Interrupting this vicious cycle might be helpful in managing patients with psoriasis.

### 6.4. Tobacco Smoking

Cigarettes contain many toxic molecules. In relation to epigenetic modification, tobacco smoking is associated with differential DNA methylation [158]. For patients with psoriasis, tobacco exposure is associated with methylation of p16^INH4a^ [159]. Torri et al. [160] have reported that the number of Th17 cells is increased in PBMCs of smokers compared to non-smokers. Tobacco smoke extract can induce Th17 differentiation and expression levels of IL-17 and IL-22, which are cytokines associated with the pathogenesis of psoriasis, in an in vitro analysis [160]. In addition, nicotinic acetylcholine receptors can modulate the function of keratinocytes through the signaling pathway [161]. Besides their expression in keratinocytes, nicotinic acetylcholine receptors are expressed in diverse immune cells, including T cells and leukemic cell lines [161], thereby nicotine exposure from tobacco smoking could disturb the immune system in patients with psoriasis.

### 6.5. Vitamin D

Vitamin D plays an important role in numerous inflammatory cutaneous disorders, including atopic dermatitis, chronic urticaria and rosacea [162,163,164]. Indeed, decreased serum levels of vitamin D in patients with psoriasis have been frequently observed [165]. The active form of vitamin D acts as a main regulator of the skin by regulating apoptosis, proliferation and differentiation of keratinocytes [166]. Moreover, vitamin D has regulatory effects on the proliferation of T cells and stimulatory effects on the propagation of Treg cells [166]. The topical formulation of vitamin D is the first line of therapies in psoriasis. Besides its anti-inflammatory effects, topical vitamin D might have therapeutic effects on psoriasis by inhibiting keratinocyte proliferation through the vitamin D receptor-associated genomic pathway and by stimulating keratinocyte differentiation via elevating intracellular calcium levels through a non-genomic pathway [166,167].

## 7. Novel Paradigm in Treatment Targets for Psoriasis

To date, a variety of treatment options is available in managing psoriasis. In general, treatment of psoriasis is based on the severity of psoriasis. The topical therapies including keratolytic, anthralin, topical steroid, vitamin D analogs and calcineurin inhibitor are used to treat the mild form of psoriasis. In patients with moderate to severe psoriasis, phototherapy or systemic therapy including retinoid, cyclosporin, methotrexate and steroid can be used. However, some agents including topical steroid, phototherapy or cyclosporin might not be suitable for sustained long-term use to manage this chronic disorder. In addition, phototherapy or systemic therapy might not be successful in some patients with moderate to severe psoriasis. In these cases, targeting more specific pathways is required for more effective treatment. With the discoveries of major molecular targets in psoriasis, the clinical trials for several biologics and small molecules are currently investigating their efficacy and safety. In this section, a brief review of the novel targeted therapies in psoriasis including anti-cytokine therapies and small molecules will be presented (Table 3).

### 7.1. Anti-Cytokine Therapies for Psoriasis

#### 7.1.1. Anti-TNF Therapy

TNF therapies have long been established for the treatment of various inflammatory disorders, including rheumatoid arthritis, ankylosing spondylitis, inflammatory bowel disease and psoriasis [183]. Among the various anti-TNF agents used in psoriasis, infliximab is a chimeric monoclonal antibody with high affinity, specificity and avidity for TNF-α [184]. In a phase III study for infliximab, about 80% of patients with moderate to severe psoriasis achieved PASI 75 by Week 6. By Week 50, 61% of these patients maintained PASI 75 [185]. Etanercept, a fully human soluble TNF receptor fusion protein, lacks signaling domains, thereby neutralizing TNF-α [186]. Adalimumab and golimumab are also fully human recombinant immunoglobulin G1 monoclonal antibodies that specifically target TNF-α. Golimumab has also been approved by the Food and Drug Administration to be used for psoriatic arthritis [169]. Recently, certolizumab pegol has been used in the treatment of psoriasis. Certolizumab pegol is a polyethylene glycol-conjugated humanized PEGylated antigen-binding fragment of the anti-TNFα monoclonal antibody. It lacks a fragment crystallizable (Fc) region. Therefore, it does not provoke an antibody-dependent cytotoxic reaction or complement activation found in other TNFα blockers [170]. PASI 75 was achieved in 83% of patients with moderate-severe psoriasis treated with 400 mg certolizumab pegol [170]. Although various clinical trial studies for these aforementioned anti-TNFα agents have shown favorable response rates in psoriasis, like other biologics, treatment efficacies of various anti-TNFα agents in psoriasis have only been demonstrated in meta-analyses. These agents were compared indirectly. Therefore, diverse head-to-head studies comparing treatment efficacies of anti-TNFα agents in psoriasis need to be conducted in the future.

#### 7.1.2. IL-12/23 Inhibitors

IL-12 and IL-23 share the same p40 subunit that binds to cell surface receptor IL-12Rβ1. Ustekinumab is a human monoclonal anti-p40 antibody that binds to the p40 subunit of IL-12 and IL-23, thereby disturbing IL-12- and IL-23-mediated cell signaling [172]. The clinical efficacy of ustekinumab in psoriasis is well known. A long-term safety study reported that the use of ustekinumab for five years in psoriasis does not increase dose-related or cumulative toxicity in patients with psoriasis [172]. Puig et al. [187] have studied the treatment efficacy among different biologics in patients with moderate to severe psoriasis and found that ustekinumab has the most favorable effects compared to adalimumab, infliximab and etanercept. Recently, the more important role of IL-23 than IL-12 for Th17 cell differentiation and survival has been accumulated. IL-23 is a heterodimer consisting of the p40 subunit, which is also observed in IL-12, and p19, distinctive for IL-23 [188]. Therefore, many types of research are selectively targeting the IL-23 p19 subunit in psoriasis. Tildrakizumab is a humanized immunoglobulin G1 monoclonal antibody targeting the IL-23 p19 subunit [173]. A phase II study of 200 mg tildrakizumab on psoriasis found that PASI 75 was achieved in 74.4% of the patients at Week 16. In addition, its treatment effect remained up to Week 52 [173]. The therapeutic potential of guselkumab, another fully human immunoglobulin G1 monoclonal antibody targeting the IL-23 p19 subunit, was tested in a recent phase III trial with 70% of patients achieving PASI 90 at Week 16 [174]. Another recent phase II trial study for risankizumab, a human immunoglobulin monoclonal antibody that selectively targets IL-23A, found that 77% of patients achieve PASI 90 after treatment with risankizumab compared to 40% of patients achieving PASI 90 after treatment with ustekinumab by Week 12 [175].

#### 7.1.3. IL-17 Inhibitor

Diverse IL-17 inhibitors are now under investigation in psoriasis. Secukinumab is a fully human immunoglobulin G1κ monoclonal antibody, while ixekizumab is a humanized immunoglobulin G4 monoclonal antibody. Both effectively neutralize IL-17A [176,177]. Brodalumab inhibits the IL-17-mediated signaling pathway in a slightly different way compared to the two aforementioned IL-17 inhibitors. Brodalumab is a human immunoglobulin G2 monoclonal antibody that can directly bind to the IL-17 receptor. It functions as an antagonist to the receptor and blocks signaling of IL-17A and IL-17F. Recently, a phase III study has been conducted to compare the clinical efficacies of brodalumab and ustekinumab [171]. Patients with moderate-to-severe psoriasis treated with brodalumab are found to have superior clinical efficacy for PASI 75 than those treated with ustekinumab [171]. Moreover, RG7624, an antibody targeting both IL-17A and IL-17F, has been reported for chronic inflammation [189]. However, no clinical trials have been conducted for this antibody targeting IL-17.

### 7.2. Targeting Small Molecules for Psoriasis

#### 7.2.1. JAK Inhibitors

In psoriasis, JAK inhibitors have emerged as promising targets for psoriasis with favorable treatment outcomes. Topical application of JAK inhibitors can inhibit lymphocyte infiltration, STAT3 phosphorylation and keratinocyte proliferation in a mouse model of contact hypersensitivity [190]. Tofacitinib is a dual inhibitor for JAK1 and JAK3. Its efficacies for diverse inflammatory disorders, including inflammatory bowel disease and psoriasis, have been recently reported. Papp et al. [182] reported that 68.8% of patients with psoriasis treated with oral 10 mg tofacitinib twice daily achieved PASI 75 at Week 28. Recently, another JAK inhibitor, ruxolitinib, was tested for psoriasis treatment. Topical application of ruxolitinib decreased the mean total lesion score in psoriasis [178]. The efficacy of baricitinib, a novel oral selective inhibitor for JAK1 and JAK2, on psoriasis was explored in a recent phase II trial with favorable results [179]. With the phase III study of baricitinib in the future, this molecule could be added as one effective novel agent for managing psoriasis.

#### 7.2.2. A3 Adenosine Receptor Agonists

A3 adenosine receptor (A3AR) is a G-protein coupled receptor regulating various intracellular signaling pathways [191]. Increased expression of A3AR has been observed in lesional inflammatory cells and PBMCs in patients with chronic inflammatory disorders, including rheumatoid arthritis, Crohn’s disease and psoriasis [191]. Inhibition of A3AR activation via A3AR agonist further downregulates the activation of NF-κB signaling pathways and promotes apoptosis of inflammatory cells [192]. CF101 is an orally-available high-affinity agonist for A3AR that exerts anti-inflammatory effects. A phase II clinical study for CF101 revealed that treatment with 2 mg CF101 resulted in progressive improvement of PASI score in patients with moderate to severe plaque-type psoriasis compared to the placebo group [180].

#### 7.2.3. IκB Kinase Inhibitor

The NF-κB signaling pathway has been recently postulated as a novel target for the treatment of psoriasis. Treatment with acetyl-11-keto-β-boswellic acid, an inhibitor of IκB kinase, can improve psoriasis disease activity score [181]. In addition, TNF-α production regulated by the NF-κB signaling pathway is decreased after treatment with IκB kinase inhibitor acetyl-11-keto-β-boswellic acid in a psoriatic mice model [181]. Therefore, targeting NF-κB signaling pathway via inhibiting IκB kinase could be used as an important strategy to manage psoriasis.

## 8. Discussion

**Figure 3:**
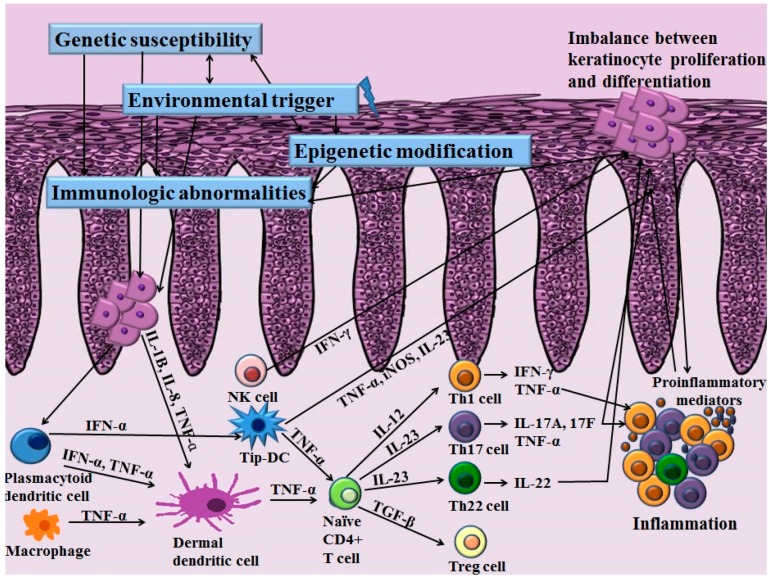
Schematic overview of effector immune cells and cytokines in the pathogenesis of psoriasis. In a genetically susceptible individual, environmental triggering factors along with epigenetic modifications affect dysregulated immune circuits in psoriasis. IFN-γ, interferon-gamma; Th, T helper cell; Treg, regulatory T cell; TGF-β, tumor growth factor-beta; TNF, tumor necrosis factor; IL, interleukin; iNOS, inducible nitric oxide synthase.

To date, many researchers have studied the molecular pathogenesis of psoriasis. The recent investigations for immunologic abnormalities in psoriasis have established the concept that the IL-23/Th17 axis is a key regulator of psoriasis. Besides T cells, diverse immunological abnormalities in cellular infiltration including keratinocytes, DCs and NK cells also play key roles in the initiation and maintenance of psoriasis (Figure 3). Imbalances in the immune system might allow the production of various inflammatory mediators and cofactors, which further affects inflammation and dysregulation in the differentiation and proliferation of keratinocytes via altered signaling pathways. Recent GWAS studies have identified various genetic components of psoriasis affecting the immune system and epidermal keratinocytes more specifically. In addition to the genetic component, the important role of epigenetic modifications in psoriasis is being currently emphasized. The interplay between genetic predisposition and epigenetic modification along with various environmental triggering factors might affect the development and persistence of psoriasis in susceptible patients. However, studies on genetic and epigenetic modifications in psoriasis are limited. Further investigations are needed to discover novel genetic risk loci and epigenetic modifications that contribute to the pathogenesis of psoriasis.

Previous management strategies for psoriasis have been focused on targeting T cells. However, many signs of progress have been made on psoriasis. The paradigm for psoriasis treatment has also shifted to targeting more specific cytokines such as TNF-α, IL-17 and IL-23. As the use of these targeting molecules will gradually increase, careful attention should be continuously paid to those newly-approved targeted molecules for psoriasis regarding its efficacy and safety data.

In conclusion, the elucidation of molecular mechanisms and pathways involved in the pathogenesis of psoriasis has yielded promising results in the field of psoriasis. However, the complex linkage hidden behind each pathogenic pathway in regards to psoriasis susceptibility genes, epigenetic modification, environmental triggering factor and immune circuits needs to be integrated. Better understanding of immunological mechanisms regulating the interplay between immune cells and keratinocytes and the identification of more upstream and downstream key cytokines and their cellular sources in psoriasis can help to identify potential novel specific targets of psoriasis in the future. To manage the recurrence of psoriatic plaque in the same area even after the treatment, future studies should focus more on identifying and targeting skin-resident immune cells in psoriasis. The additional genetic phenotype studies estimating the treatment responses among various targets might bring more potential and novel tailored therapy to the patient. Thorough understanding of the molecular pathogenesis of psoriasis could bring promising results not only in the field of psoriasis, but also for other chronic inflammatory diseases that might possess similar cellular and molecular pathogenic networks in their pathogenesis.

## Figures and Tables

**Table 1:** Genetic loci for psoriasis susceptibility.

Gene Loci	Chromosomal Locus	SNP	Function of the Protein	Reference
**Antigen presentation**				
*HLA-C*	6p21	rs12191877	Antigen presentation; MHC class I	[74]
*ERAP1*	5q15	rs27432	Peptidase to trim peptides for binding to MHC 1 antigen presentation	[83]
**Th1 or Th17 cell differentiation/regulation**				
*RUNX3*	1p36	rs7536201	Transcription factor regulating Th1 and memory T-cell differentiation	[84]
*SOCS1*	16p13	rs367569	Th17 cell differentiation	[86]
*IL-12B*	5q31	rs4379175 rs3213094	Encoding p40 subunit of IL-23 and IL-12 and promoting Th1 cell differentiation	[87,100]
*IL-23R*	1p31	rs2201841 rs11209026	Encoding IL-23 receptor subunit and inducing TNFα-dependent epidermal hyperplasia	[88,91]
*TRAF3IP2*	6q21	rs13210248, rs33980500	Modulation of IL-17 signaling and affects the NF-κB signaling	[79]
*IL23A*	12q13	rs2066808	Encoding p19 subunit of IL-23	[88]
**NF-κB signaling**				
*CARD14*	17q25	rs11652075	Activation of NF-κB signaling	[94]
*REL*	2p16	rs702873	Involved in NF-κB signaling	[95]
*TNIP1/ANXA6*	5q33	rs2233278	Modulation of NF-κB signaling	[96]
*TNFAIP3*	6q23	rs610604	Modulation of NF-κB signaling	[88]
*UBE2L3*	22q11	rs4821124	Regulating NF-κB signaling	[84]
*CARM1*	19p13	N/A	Coactivator for NF-κB signaling	[61]
*NFKBIA*	14q13	rs8016947	Inhibition of NF-κB signaling	[91]
*FBXL19*	16p11	rs12445568	Inhibition of NF-κB signaling	[97]
**IFN signaling**				
*IFIH1*	2q24	rs17716942	RIG-like helicase; antiviral receptor	[90]
*DDX58*	9p12	rs11795343	Innate RIG-1 antiviral signaling	[61]
*RNF114*	20q13	rs1056198	Innate antiviral signaling; E3 ubiquitin ligase	[91]
*IL-28RA*	1p36	rs4649203	IFN signaling , IL-29 receptor subunit	[92]
*TYK2*	19p13	rs12720356	Involved in IFN signaling	[83]
*EXOC2*	6p25	rs9504361	Promotes production of type 1 IFNs in response to intracellular DNA	[93]
*ELMO1*	7p14	rs2700987	Enhances toll like receptor mediated IFN-α production	[93]
**Epidermal keratinocytes**				
*LCE3B/LCE3C*	1q21	rs6677595	Structural protein for keratinocytes	[101]
*LCE3D*	1q21	rs4112788	Regulates terminal differentiation of epidermal keratinocytes	[91]
*LCE1C*	1q21	rs6701216	Structural protein for keratinocytes	[98]
*GJB2*	13q12	rs3751385	Connexin 26	[102]

**Table 2:** Various microRNAs with perturbed expression in psoriasis.

miRNAs	Expression	Target Genes	Possible Mechanism of Action on Psoriasis	References
miR-21	Upregulated	*TIMP3*, *TACE/ADAM17*	Activation of TNF-α signaling, suppression of apoptosis in activated T cells	[126,127]
miR-31	Upregulated	*STK40*, *FIH-1*, *ppp6c*, *EMP-1*	Proliferation and differentiation of keratinocytes Modulation of TGF-β1 and NF-κB signaling	[128,129,130]
miR-136	Upregulated	*PPP2R2A*	Modulation of TGF-β1-associated keratinocyte proliferation arrest	[131]
miR-143	Upregulated	*SLC26A4*	Recruitment of neutrophils and monocytes from peripheral blood	[132]
miR-146	Upregulated	*EGFR*, *FERMT1*	Keratinocyte proliferation	[120,133]
miR-155	Upregulated	*CTLA-4*	Regulation of T cell activation, involved in development of dendritic cells and Treg cells	[134,135]
miR-203	Upregulated	*SOCS-3*, *STAT3*, *SOSC-6*	Suppression of SOCS-3-dependent signaling, regulation of keratinocyte proliferation and differentiation via STAT3	[136,137]
miR-221/2	Upregulated	*TIMP3*	Degradation of TIMP3, regulation of keratinocyte growth and apoptosis	[138]
miR-223	Upregulated	*GLUL*, *SMAD3*	Modulation of leukocyte chemotaxis	[132]
Has-miR-99a	Downregulated	*IGF-1R*	Modulation of keratinocyte proliferation and differentiation	[125,139]
miR-125b	Downregulated	*FGFR2*	Modulation of keratinocyte proliferation	[121]
miR-138	Downregulated	*RUNX3*	Modulation of Th1/Th2 balances on CD4^+^ T cells	[140]
miR-424	Downregulated	*MEK1*, *Cyclin E1*	Modulation of MEK1 and cyclin E1 dependent keratinocyte proliferation	[135]

**Table 3:** Summary of novel targeted therapies in psoriasis.

Targets	Drug	Mode of Action	Route of Administration	Ref.
**Biologics**				
TNF-α	Infliximab	Chimeric anti-TNF-α mAb	IV	[74]
	Adalimumab	Fully human anti-TNF-α mAb	SC	[83]
	Etanercept	Human soluble TNF-α receptor	SC	[168]
	Golimumab	Fully human anti-TNF-α mAb	SC	[169]
	Certolizumab pegol	Humanized PEGylated antigen binding fragment of anti-TNF-α mAb	SC	[170]
IL-12/23	Ustekinumab	Fully human anti-IL-12/23 p40 mAb	SC	[171,172]
IL-23	Tildrakizumab	Fully human IgG1 anti-IL-23 p19 mAb	SC	[173]
	Guselkumab	Fully human IgG1 anti-IL-23 p19 mAb	SC	[174]
	Risankizumab	Fully human anti-IL-23 p19 mAb	SC	[175]
IL-17	Ixekizumab	Humanized IgG4 anti-IL-17 mAb	SC or IV	[176]
	Secukinumab	Fully human IgG1κ anti-IL-17 mAb	SC or IV	[177]
	Brodalumab	Fully human IgG2 anti-IL-17 receptor mAb	SC	[171]
	Ruxolitinib	Selective inhibitor of JAK1 and JAK2	Topical	[178]
	Baricitinib	Selective inhibitor of JAK1 and JAK2	Oral	[179]
A3AR	CF101	High affinity agonist for A3AR	Oral	[180]
IκB kinase	Acetyl-11-keto-β-boswellic acid	IκB kinase inhibitor	Topical	[181]
**Small molecules**				
JAK	Tofacitinib	Selective inhibitor of JAK1 and JAK3	Oral, Topical	[182]

Abbreviation: Ref., reference; TNF, tumor necrosis factor; mAb, monoclonal antibody; IV, intravenous; SC, subcutaneous; IL, interleukin; Ig, immunoglobulin; JAK, Janus kinase; A3AR, A3 adenosine receptor.

## Abbreviations

A3AR	A3 adenosine receptor
CCR	Chemokine receptor
DC	Dendritic cell
EDC	Epidermal differentiation complex
EGF	Epidermal growth factor
ERAP1	Endoplasmic reticulum aminopeptidase 1
FoxP3	Forkhead/winged helix transcription factor 3
GWAS	Genome-wide association studies
HDAC	Histone acetyltransferases and histone deacetylase
HLA	Human leukocyte antigen
IFN	Interferon
IL	Interleukin
iNOS	Inducible nitric oxide synthase
JAK	Janus kinase
KGF	Keratinocyte growth factor
KIRs	Killer immunoglobulin-like receptors
LncRNA	Long noncoding RNA
MHC	Major histocompatibility complex
miRNA	MicroRNA
NF-κB	Nuclear factor-kappa B
NK	Natural killer
PASI	Psoriasis Area and Severity Index
PBMC	Peripheral blood mononuclear cells
pDC	Plasmacytoid dendritic cells
PGRP	Peptidoglycan recognition proteins
PSORS	Psoriasis susceptibility locus
RIG-1	Retinoic acid inducible-gene 1
STAT	Signal transducer and activator of transcription
Th	T helper
Treg	T regulatory
Tip-DC	TNF-α and iNOS-producing DC
TNF	Tumor necrosis factor
